# Contextualizing and optimizing novel strategies to improve the latent TB continuum of care: Insights from people living with HIV and health care providers in Brazil

**DOI:** 10.1371/journal.pgph.0001251

**Published:** 2023-01-04

**Authors:** Isadora Salles, Paula Travassos, Renata Spener-Gomes, Ana Paula Loch, Valeria Saraceni, Lilian Lauria, Solange Cavalcante, Jamile Garcia de Oliveira, Alexandra Brito de Souza, Allyson Guimarães Costa, Sumire Sakabe, Roberta Schiavon Nogueira, Lelia H. Chaisson, Silvia Cohn, Leda Fatima Jamal, Jose Valdez Ramalho Madruga, Marcelo Cordeiro-Santos, Barbara Castro, Danielle Portella Ferreira, Christopher J. Hoffmann, Jonathan E. Golub, Betina Durovni, Deanna Kerrigan

**Affiliations:** 1 Department of Medicine, Johns Hopkins University School of Medicine, Baltimore, Maryland, United States of America; 2 Secretaria Municipal de Saúde, Rio de Janeiro, Brazil; 3 Gerência de Micobacteriologia, Fundação de Medicina Tropical Dr. Heitor Vieira Dourado, Manaus, Brazil; 4 Programa de Pós-Graduação em Medicina Tropical, Universidade do Estado do Amazonas, Manaus, Brazil; 5 Universidade Federal do Amazonas, Manaus, Brazil; 6 Centro de Referência e Treinamento DST/Aids, Secretaria de Estado da Saúde de São Paulo, São Paulo, Brazil; 7 Fundação Oswaldo Cruz (Fiocruz), Rio de Janeiro, Brazil; 8 Escola de Enfermagem de Manaus, Universidade Federal do Amazonas, Manaus, Brazil; 9 Division of Infectious Diseases, Department of Medicine, University of Illinois at Chicago, Chicago, Illinos, United States of America; 10 Universidade Federal do Rio de Janeiro, Rio de Janeiro, Brazil; 11 Department of Prevention and Community Health, Milken Institute School of Public Health, Washington, District of Columbia, United States of America; Universitas Sebelas Maret Fakultas Kedokteran, INDONESIA

## Abstract

Tuberculosis (TB) causes 1 in 3 deaths among people living with HIV (PLHIV). Diagnosing and treating latent tuberculosis infection (LTBI) is critical to reducing TB incidence and mortality. Blood-based screening tests (e.g., QuantiFERON-TB Gold Plus (QFT+)) and shorter-course TB preventive therapy (TPT) regimens such as 3HP (3 months weekly isoniazid-rifapentine) hold significant promise to improve TB outcomes. We qualitatively explored barriers and solutions to optimizing QFT+ and 3HP among PLHIV in three cities in Brazil. We conducted 110 in-depth interviews with PLHIV, health care providers (HCP) and key informants (KI). Content analysis was conducted including the use of case summaries and comparison of themes across populations and contexts. LTBI screening and treatment practices were dependent on HCP’s perceptions of whether they were critical to improving TB outcomes. Many HCP lacked a strong understanding of LTBI and perceived the current TPT regimen as complicated. HCP reported that LTBI screening and treatment were constrained by clinic staffing challenges. While PLHIV generally expressed willingness to consider any test or treatment that doctors recommended, they indicated HCP rarely discussed LTBI and TPT. TB testing and treatment requests were constrained by structural factors including financial and food insecurity, difficulties leaving work for appointments, stigma and family responsibilities. QFT+ and 3HP were viewed by all participants as tools that could significantly improve the LTBI cascade by avoiding complexities of TB skin tests and longer LTBI treatment courses. QFT+ and 3HP were perceived to have challenges, including the potential to increase workload on over-burdened health systems if not implemented alongside improved supply chains, staffing, and training, and follow-up initiatives. Multi-level interventions that increase understanding of the importance of LTBI and TPT among HCP, improve patient-provider communication, and streamline clinic-level operations related to QFT+ and 3HP are needed to optimize their impact among PLHIV and reduce TB mortality.

## Background

Advances in antiretroviral therapies have significantly improved the survival of people living with HIV (PLHIV) [1], yet tuberculosis (TB) remains the leading cause of death among PLHIV worldwide, causing one in three HIV deaths in 2020 [2]. Brazil is among the top 10% of countries with the greatest TB/HIV burdens [3], with 8.4% of the total of 66, 819 newly diagnosed TB cases occurring among PLHIV in 2020 [4].

Treating PLHIV with latent TB infection (LTBI) is a key component of global and national recommendations for TB control [2,5]. Brazilian TB guidelines recommend TB preventive therapy (TPT) for all PLHIV with a CD4 count <350 cells/mm^3^. For those with a CD4 count ≥350 cells/mm^3^, LTBI screening with the tuberculin skin test (TST) or Interferon-Gamma Release Assay (IGRA) is recommended annually, with TPT for those who test positive [5,6]. However, many eligible patients are not screened for LTBI with TST, resulting in suboptimal delivery of TPT [7,8]. IGRAs are a promising alternative to TST for LTBI screening in PLHIV. IGRAs have higher sensitivity in PLHIV, reducing the number of false-negatives, and use *Mycobacterium tuberculosis* (*M*.*tb)* specific antigens, minimizing the occurrence of cross-reactivity [9]. IGRAs can be performed in one clinic visit, eliminating the need for patients to return to the clinic for a test reading, as is the case with the TST. QuantiFERON-TB Gold Plus (QFT+) is a latest generation of IGRA used for LTBI screening. IGRA tests were first approved for use in Brazil in 2020. Yet, QFT+ is not currently widely available for routine use through the country’s public health network [10].

In addition to LTBI screening, TPT initiation and completion remains a significant challenge, even when TPT is provided free of charge [11]. The long treatment period and potential for adverse drug reactions with the current 6- or 9-month daily isoniazid preventive therapy (IPT) regimen for PLHIV are noted disincentives to treatment completion [12]. The World Health Organization [13] has endorsed a 3-month, once-weekly isoniazid plus rifapentine (3HP) TPT regimen, which has been shown to have higher treatment completion rates and lower levels of toxicities than IPT [14–17]. In July 2021, 3HP was adopted into Brazilian national guidelines as a recommended regimen for LTBI treatment. However, rollout of 3HP remains limited in Brazil [18] and other high burden countries such as Uganda [19], China [20], and Pakistan [21]. Exploring the context of implementing these new technological advances is essential for streamlining the rollout and success use of QFT+ and 3HP within routine clinical care, particularly in medium and high TB burden countries. To address these gaps, we qualitatively explored the context, barriers and facilitators to implementing QFT+ and 3HP in Brazil.

## Methods

PREVINE is a multi-center implementation study that aims to improve LTBI screening and TPT uptake among PLHIV through implementation of QFT+ paired with routine CD4 and viral load measurements at clinics in three cities in Brazil: Rio de Janeiro, Manaus, and São Paulo. We conducted the qualitative research presented here between December 2019 and February 2020, prior to the initiation of PREVINE intervention activities, to help tailor study components for optimal intervention implementation.

In total, 110 in-depth interviews (IDIs) were conducted, including with PLHIV (n = 40), their health care providers (HCPs) including physicians and nurses (n = 40) and key informants (KIs), including individuals who helped to manage and coordinate clinic HIV/TB services, but were not involved in direct patient clinical care (n = 30). Ten interviews with PLHIV, HCPs, and KIs were conducted in each city, including 2 clinics in Rio de Janeiro, 1 clinic in São Paulo, and 1 clinic in Manaus. Participants were purposively sampled to achieve a balance of the sample in terms of gender (half men and half women) and to generate diversity in terms of socio-economic status among participating PLHIV. Study investigators from each site helped identify and recruit potential PLHIV participants, and relevant HCPs and KIs.

A semi-structured guide using open-ended questions related to the context of the proposed interventions was used to facilitate and support discussion in the interviews. We also employed free listing and ranking techniques in relation to these strategies across population groups within IDIs [22]. The interviews were audio-recorded and transcribed. Thematic content analysis was conducted [23]. We first created case summaries of each IDI. The two independent team members conducting analysis met regularly to compare emergent themes. Case summaries from individual interviews were then synthesized across population groups. Salient themes were extracted and compared across populations and geographic contexts, and then shared with additional study investigators, including those on the ground with first-hand knowledge of these dynamics unfolding in the field to ensure validity of themes. Global themes identified across settings were then extracted and illustrative quotes selected to represent the key findings. Notetaking was used throughout the research process to enhance rigor encouraging researcher reflexivity, insights on their own biases and rationale for decision-making as the study progressed [24].

Written informed consent was obtained from each study participant. All interviews were conducted in a private setting at the respective clinic. The study protocol was approved by the Institutional Review Board (IRB) of the Johns Hopkins School of Medicine (FWA00005752), National Research Ethics Commission (Comissão Nacional de Ética em Pesquisa (CONEP)) in Brazil, and relevant local IRBs.

## Results

### Sample characteristics

Forty interviews were conducted with PLHIV at four clinics across Rio de Janeiro, São Paulo, and Manaus, including 20 women and 20 men. The mean age of PLHIV was 46.1 years (range 26–70). The mean years of education was 10.9 years (range 4–18) and most were employed in the service industry (e.g., waiter, housekeeper). Patients had been living with HIV for a mean of 12.9 years (range 1.5–37) and had spent a mean of 10.3 years (range 0.5–32) in HIV care at the clinics from which they were recruited. All patients were currently on antiretroviral therapy (ART), with a mean of 10.4 years (range 0.6–30) on ART. Eight (20%) patients had a history of TB and three (7.5%) reported previously taking TPT (Table 1).

**Table 1:** Sociodemographic characteristics of the sample (N = 110).

**People living with HIV (n = 40)**	
Characteristic	N (%) Mean (range)
Gender Female Male	20/40 (50%) 20/40(50%)
Age	46.1 (26–70)
Education (years)	10.9 (4–18)
Years livings with HIV	12.9 (1.5–37)
Years receiving HIV care at clinic where recruited	10.3 (0.5–32)
Years on ART	10.4 (0.6–30)
History of TB	8/40 (20%)
Previous TPT	3/40 (7.5%)
**Healthcare Providers (n = 40)**	
Gender Female Male	30/40 (75%) 10/40 (25%)
Role Physician Nurse Other	12/40 (30%) 13/40(32.5%) 15/40 (37.5%)
Years working at study clinics	10.4 (0.5–28)
Years working in TB and/or HIV overall	13.8 (2.8–28)
**Key Informants (n = 30)**	
Gender Female Male	21/30 (70%) 9/30 (30%)
Role Physician Nurse Other	18/30 (60%) 7/30 (23.3%) 5/30 (16.7%)
Years working at study clinics	17.6 (0.6–42)
Years working in TB and/or HIV overall	18.6 (4–32)

Forty interviews were conducted with HCPs; the majority were women (30 out of 40; 75%). HCPs were primarily employed as physicians (30%) or nurses (32.5%) at the participating clinics. On average, providers had spent 10.4 years working at study clinics (range 0.5–28) and a mean of 13.8 years (range 2.8–28) working in the areas of TB and/or HIV overall.

Thirty key informant interviews were conducted. The majority of KIs were women (21 out of 30; 70%). KIs were also primarily physicians (60%) or nurses (23.3%), and held additional supervisory roles at the clinic or were involved in managing and coordinating TB/HIV programs within the clinic. KIs had spent a mean of 17.6 years (range 0.6–42) working at the study clinic, and had worked in the area of TB and/or HIV overall for a mean of 18.6 years (range 4–32).

### Context and challenges of introducing novel LTBI screening and treatment strategies

**Fig 1:**
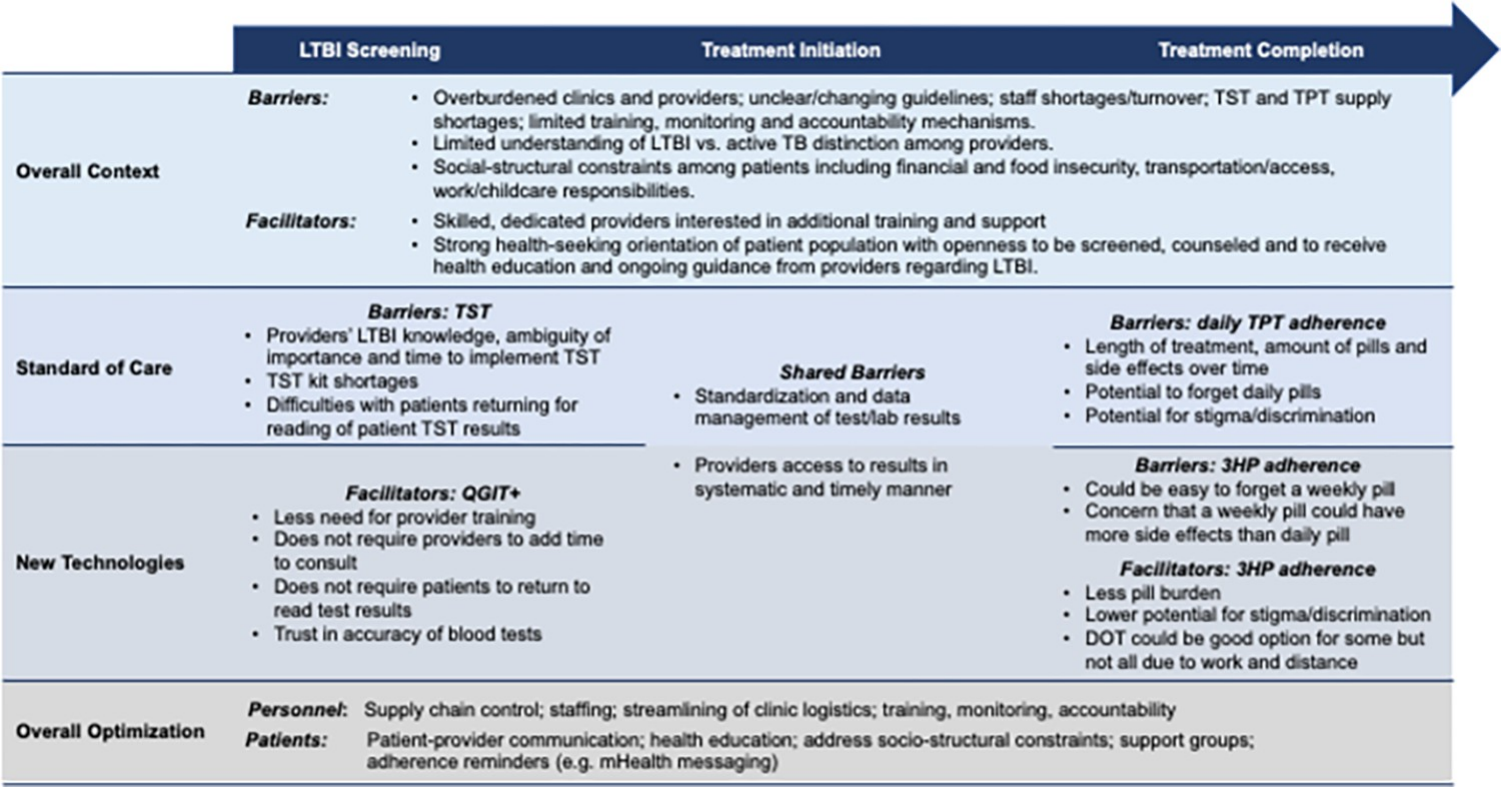
Clinic context, barriers, facilitators, and optimization strategies to improve the LTBI Care Continuum among PLHIV in Brazil via new technologies.

The context of introducing LTBI screening and treatment strategies into health systems and clinic dynamics sets the stage for provider and patient attitudes and behaviors and is a key component to consider when introducing novel technologies. These factors are detailed below and depicted in *Fig 1*.

#### LTBI screening

HCPs and KIs were generally supportive of current Brazilian national TB policies and protocols, which involved annual screenings for PLHIV with TST and TPT for those with a positive TST, or with CD4 count ≥350 cells/mm^3^. However, many providers reported that a primary motivation to screen for TB was to identify and treat active TB rather than to screen and treat LTBI. In turn, many providers indicated that they had not fully integrated annual LTBI screenings and prescribing TPT for those diagnosed with LTBI into their clinical routines. Patients interviewed mirrored this finding, reporting that LTBI was rarely discussed during consults. Providers noted that they ordered a TST if their patients ever mention experiencing TB symptoms or came in contact with someone recently diagnosed with TB. A key informant interviewed from São Paulo described this issue.

“*I think that this screening [LTBI]*, *although it is part of the recommendations*, *it is neglected by a good portion of professionals*. *We see a very large number of services where you don’t ask [about symptoms or TB contact]*, *if the patient is fine [no obvious TB symptoms]*, *you don’t ask*. *It has become a situation that you ask about if there is something serious going on…We recently reviewed medical records and noticed that many doctors forgot to screen for latent TB infection*.*”—Key Informant*, *Male*, *São Paulo*

In addition to the perceived need for LTBI screening, HCPs and KIs also commented on the logistical and clinic operational challenges associated with TST to screen for LTBI. These challenges included the need to return to have the TST read on a separate occasion and purified protein derivative (PPD) skin test shortages causing consistent TST use to fall out of practice. Additional challenges described by HCPs and KIs included staffing turnover, providers moving from clinic to clinic, and HCP shortages also made providers feel rushed in their consultations with patients. Additionally, policy level factors such as changing national guidelines created confusion among providers when trying to keep up with which patients should have a TST done, and which could be prescribed TPT without a positive screening test.

“*Well*, *it’s a bit confusing for us*, *because for a while it was the Ministry’s strategy*, *since there wasn’t any PPD*, *they ended up indicating that everyone who met a certain [CD4] criteria should get TPT*, *regardless of the PPD test*. *Not today though*, *since PPD is back*, *now we are using the PPD test for all patients with HIV*.*”—Health Care Provider*, *Female*, *Manaus*

#### Treatment initiation

When providers did screen for LTBI, they were often hesitant to prescribe TPT because it was seen as complicated for patients to manage due to the long length of treatment. In addition to the length of treatment, HCP and patients identified the large quantity of pills, potential for adverse reactions, and lack of symptoms to associate with the need for the medication as the greatest barriers to TPT adherence and treatment completion.

“*Look*, *it works*, *but we know that the issue of adherence is a little complicated because patients often cannot understand the idea of prophylaxis*. *They think that it is already a treatment and*, *the length of the treatment interferes with adherence…The patient must understand what is happening and we know that*, *sometimes*, *it is so busy [in the clinic]*, *that we sometimes lose this patient in the middle of treatment*. *So*, *it is effective*, *but if we could optimize the length*, *I think it would be much better for patients*.*”—Health Care Provider*, *Female*, *Manaus*

TPT adherence was described by providers as particularly challenging for patients that have other priorities like accessing food or have poor health literacy, which was thought to make them hesitant to take another medication in addition to ART as part of their daily routine, especially since TPT medications can cause side effects and adverse reactions.

“*It’s not everybody’s reality*, *you know*? *But the reality of many people here*, *for example*, *is poverty*, *the poverty is so great*, *so great*, *homeless people*, *our homeless people are all infected with TB*, *you know*? *And HIV*, *many of them*. *And then*, *the person has nothing to eat*, *do you really think they are going to take medicine*? *I think there should be a social strategy behind it…Maria José [pseudonym]*, *for example*, *arrived with serious tuberculosis*, *she had nothing to eat*, *do you think she will take a medication that will make her super nauseous*? *They don’t even have anything to eat*, *man*.*”—Health Care Provider*, *Female*, *Manaus*

#### Maintaining willingness to test and treat LTBI among logistical and operational challenges

Given this combination of attitudinal, logistical, operational and structural factors, many providers were not convinced that screening for LTBI and prescribing TPT for those with a positive result was worthwhile. HCP were often eager for solutions to make their patients’ lives more comfortable and to not lose their patients’ willingness to have tests done by ordering tests or prescribing medications that would greatly interfere with their daily lifestyle and routines.

Though many patients had never heard of LTBI or isoniazid preventive therapy, many expressed willingness to get any test or treatment their doctors say they should, but they were often balancing that with the structural constraints of their daily lives, particularly as it related to the need to maintain employment and manage other responsibilities such as caring for families.

“*Yeah*, *the way I think*, *if my doctor tells me that the exam has changed*, *we won’t do the PPD anymore*, *we’ll collect your blood and test it from there*, *I trust her*.*” - 33-year-old Patient*, *Male*, *Rio de Janeiro*

Many prioritized their health, noting they would adapt their personal circumstances if their health depended on it.

“*Yes*, *whether we like it or not*, *we have to accept it [getting a QFT+ test]*, *because health comes first*, *right*? *Work comes in second place*…*Without health we are nothing*, *this flesh here rots*, *that’s it for us*.*” - 35-year-old Patient*, *Female*, *Rio de Janeiro*

#### Technological solutions to address LTBI screening and treatment challenges: QFT+ and 3HP

Despite challenges within the health systems and clinical context regarding LTBI screening and treatment, providers, patients, and key informants indicated support for new technologies and generated important ideas about how to optimize their use in the clinical space as seen below and highlighted in *Fig 1*.

#### LTBI screening using QFT+

There was a nearly universal consensus among all study participants that using QFT+ for screening at routine CD4 and viral load blood draws among PLHIV was logistically advantageous over screening with TST. Patients expressed that they are accustomed to having blood drawn since most undergo such assessments approximately every 6 months. Patients liked that they could get the test done at the same time as their regular visit and would not need to return to clinic a few days later, avoiding time off from work and time away from other responsibilities (e.g., family), and additional costs of going back to the clinic which in many cases was a far distance from home.

“*I think it would be better*, *because blood tests are already in my routine*, *right*. *I have them done at least three times a year*. *So*, *if I could include it [LTBI screening] then*, *that’s great*, *isn’t it*? *It’s one less test to have done*. *In short*, *it’s easier*. *Also*, *because for the PPD*, *you get it done then need to return for the reading days later*. *I don’t live so close to here [the clinic]*.*” - 33-year-old Patient*, *Male*, *Rio de Janeiro*

Health professionals agreed that offering a more comfortable and streamlined LTBI screening process, which would interfere less with patients’ daily routines, and lessen the chances of patients being lost in the diagnostic process, would encourage clinicians to order these tests.

“*I think it would be excellent*. *I really like working with this idea of “the opportune moment”*, *we know that so many patients here are doing well*, *because they have a life to take care of*, *they work*, *they have a normal life*, *so they end up not having the availability to keep leaving work to come in all the time*. *So*, *if we manage to combine everything into one service*, *all in one collection day*, *the less they come into the clinic*, *the better their adherence will be…Also*, *for patients with poor adherence*, *because if you miss that opportunity while he is here at the clinic to do CD4/viral load testing*, *it is a missed opportunity because you don’t know if and when they will be back*.*”*

- Health Care Provider, Female, São Paulo

Providers and program managers also reported that they liked that QFT+ would not require any additional training for technicians, since many mentioned it was difficult to promote training in TST administration among health personnel. Many HCPs felt that they were already very busy with their current responsibilities and did not want to add any additional work burden. Additionally, there would be less subjectivity in reading test results than with the TST, since QFT+ goes through the laboratory instead of requiring a visual inspection of the application site on the arm, as is the case with TST.

Many patients also relayed that they saw a blood test as a more standard screening tool for any kind of disease, and would be more confident about results from a blood test than a skin test.

“*Today you can detect so many things through blood*, *right*? *So*, *I think it would be better if it were by blood…because it’s easier to actually confirm that a person has a disease through blood*, *right*? *I think that skin one is strange*. *It’s more practical …you can detect diseases that sometimes we doubted we even had*.*” - 51-year-old Patient*, *Female*, *Manaus*

#### Treating LTBI using 3HP

3HP was seen as a significant improvement for the patient experience compared to the current 6- or 9-month isoniazid regimen from the patient, HCP, and program manager perspectives. Providers and program managers noted that reducing the length of treatment and number of pills would significantly improve the patient experience and encourage adherence. The weekly dose was also seen as beneficial in terms of reducing the potential for stigma among patients who take their medications secretly to conceal their HIV and/or TB diagnoses.

“*The positive aspects*: *it will reduce the amount of medication*, *especially for those here who take so many medications*. *Because we say that the biggest challenge is for you to align the schedules so that the patient can have a life outside without depending so much on those medication schedules*. *Because many work*, *they don’t want anyone at work to find out*. *We had a pregnant woman with tuberculosis*, *that she took the medication hidden*, *because she did not want the work people to discover that she had HIV and tuberculosis*. *So when there were a lot of people around*, *she didn’t take the medication*. *So I think that if you reduce the amount of medication and reduce these hours*, *it would facilitate their adherence*. *I only see advantages*.*”—Health Care Provider*, *Female*, *São Paulo*

Participants noted some potential challenges with a weekly regimen. The most mentioned challenge of 3HP is that a weekly dose may be easier to forget than a daily dose. Because of this, both patients and providers mentioned that a weekly pill with 3HP would require patients to be more organized with their medications.

“*It’s obvious that one positive aspect is the shortened length of treatment*, *another positive aspect is reducing the dose*, *of course*. *And the negative aspect too*? *It’s very easy to forget*.*”—Key Informant*, *Female*, *São Paulo*

Despite this challenge, many patients mentioned that they already regularly take several medications at different hours of the day and that they have established reminder strategies, such as setting alarms or leaving notes on their fridge, that could be easily adapted to include a weekly pill if they were diagnosed with LTBI.

“*So*, *for me*, *adding another medication into my routine isn’t a big deal*. *But obviously if I can take the medication once a week instead of daily*, *of course it would be better*.*” - 33-year-old Patient*, *Male*, *Rio de Janeiro*

Although patients expressed willingness to take any medications that their doctors prescribed, some feared that the TPT would cause adverse reactions. Related to this was a concern that 3HP would be a stronger dose than IPT, since it is given weekly rather than daily, which could potentially increase the risk of adverse reactions.

“*But then it would be very strong*, *right*? *Oh*, *I don’t know*. *Would the person react well to a medication that strong*? *… And then I don’t know if I could take a very strong medication*, *because there are people who say that they get dizzy*, *that they feel sick…” - 42-year-old Patient*, *Female*, *Rio de Janeiro*

Conversely, many participants saw the less frequent dosing as an opportunity for the body to recover from any adverse reactions they did experience and ultimately lessen the amount of chemicals in their body that would result in adverse reactions.

“*I would prefer this one pill a week*, *because it’s less chemicals in the body*, *right*? *And being once a week*, *maybe you can even recover better from the side effects that the meds will give you*. *But I think it’s very strange*, *this idea of three months or the longer one*, *because if I saw myself with this problem*, *I would take it regardless*, *even if it was a year or more you need to take it for*. *Because it is impossible for you to live well with this condition*, *so you need to treat it*. *I can’t understand how a person that may have this latent virus*, *or has the active virus*, *and doesn’t adhere [to treatment]*, *knowing that they are HIV positive*. *Then it’s because of a huge lack of information*.*” - 35-year-old Patient*, *Male*, *Rio de Janeiro*

A majority of patients who were hesitant about side effects said they would continue on the medication as long as they could discuss ways to mitigate potential side effects with their HCPs.

### Optimizing implementation of QFT+ and 3HP

While there was strong agreement among all participants that QFT+ and 3HP have the potential to address many of the challenges with current LTBI screening and treatment protocols, these innovations had their own challenges, necessitating support to optimize their use.

#### Health education to address the need for information on LTBI screening and treatment

In relation to both QFT+ and 3HP, free list analysis showed that improved health education about LTBI in general and specifically related to QFT+ and 3HP was the most salient item needed to optimize implementation across all participant groups and settings. Health education topics noted included information about risk of LTBI among PLHIV, the significance of LTBI screening and treatment for prevention, and the importance of adherence to treatment.

#### Ensuring streamlined flow of logistics in clinics to prevent increased stress and work burden

HCPs reported that improvements to LTBI screening infrastructure, sufficient staffing, and high-quality training for HCPs involved in the QFT+ process were needed to allow for a streamlined flow of QFT+ testing logistics and to prevent any increases in the current workload.

HCPs often viewed processes for LTBI screening and treatment as cumbersome, including ordering forms and results notifications systems, making the screening and treatment process confusing and time consuming for both providers and patients. HCPs reported that the extensive number of steps involved in screening were a disincentive to screen and treat LTBI. HCPs noted that, if QFT+ could be ordered using the CD4 and viral load blood draw order form, it would help facilitate use of QFT+. Additional suggestions included an option for QFT+ in the electronic medical records system, where available, to be ordered with a click, making it easier for providers to remember to order the test, while encouraging them to screen for LTBI more often and more consistently.

“*It could be like a packet*, *because many doctors have difficulty when ordering multiple tests*… *On paper or in the system*, *they don’t want to do any extra work*. *So*, *you could bundle it*: *every 6 months*, *the doctor will order the viral load*, *CD4 and tuberculosis tests*, *understand*? *Inserting it in a single click*, *they click it*, *it would get done*, *I think it would be easier*.*”—Health Care Provider*, *Female*, *Manaus*

#### Ensuring LTBI screening supplies and making results available quickly

Given the history of TST supply stockouts, ensuring a steady supply of QFT+ materials stood out as a crucial step to optimizing the implementation of QFT+ and incorporating the new test into routine practice. Additionally, establishing both a streamlined and rapid logistical flow related to HCPs reported that LTBI screening was key to preventing diagnostic delays. Many noted that it was crucial to ensure that there were clear laboratory protocols to not only process test samples quickly so that patients can receive their result quickly but also making sure that HCPs received results in an easily accessible manner to be able to act on them in a timely manner.

#### Discussing treatment plan with patient

Because providers often reported being rushed during consultations, patients sometimes left with a diagnosis or TPT prescription without thoroughly understanding their diagnosis or treatment plan. Patients echoed this sentiment and expressed that providers often did not take or have the time to explain things well. Related to this was a desire from patients to have more clear communication, accessible information, and health education.

Improvements to the current landscape to optimize 3HP that were suggested by HCPs also included discussing the treatment timeline, informing patient on next steps if they forget to take a dose, learning how to mitigate any adverse reactions, and allowing time for patients to ask questions and express any concerns that they may have about their treatment to their provider.

#### Individualize treatment plan

Many participants emphasized that although new technological advances in LTBI screening and treatment may help patients improve their clinical outcomes, it will be critical to examine both the biomedical and social context of patients’ lives and any extenuating circumstances that keep patients from adhering to treatment and care.

“*I think that perhaps our greatest difficulty*, *our greatest challenge is*: *in addition to having this intervention*, *offering medication*, *is trying to think of other ways to help*, *which go beyond what happens here*, *go beyond our piece*, *which is the biological part*, *that isn’t all that’s going on*. *So the fragility is there*, *right*? *We take care of our piece very well*, *we are trying to make a very timely diagnosis*, *before he gets an active infection*, *but what else*?*”—Health Care Provider*, *Female*, *São Paulo*

Participants suggested individualizing treatment plans by establishing reminder strategies to engage patients in their treatment decisions. Many were in favor of using text or phone call reminders, while others suggested alarm clock reminders, others proposed allowing patients to pick what day of the week they wanted to take the 3HP medication. These strategies were seen as beneficial for improving treatment adherence.

#### Utility of DOT for patients needing additional support

A vast majority of providers believed directly observed therapy (DOT) for 3HP would be unnecessary for a majority of their patients, that reminder strategies would be sufficient for a majority of cases. Yet, several emphasized the utility of DOT in a minority of cases for patients who have difficulty managing their treatment alone. The weekly dose with 3HP, compared to a daily dose, was seen as lending better to DOT for patients who need additional support. Because of this, 3HP would be an opportunity for clinics to implement DOT programs more easily for patients who would benefit most from it. Patients were also supportive of DOT, particularly among those who had easy access to the clinic.

#### Monitoring adherence using support groups and mobile health (mHealth)

HCPs often expressed feeling overwhelmed with their current workload and believed that it would be difficult for them to take on the additional responsibility of monitoring patients receiving 3HP. There would need to be systems-level improvements to current TPT monitoring. In response, many suggested using multidisciplinary teams to improve TPT monitoring practices. The use of multidisciplinary teams would also provide patients with multiple opportunities to engage with health professionals and discuss any extenuating circumstances that hinder their ability to adhere to treatment.

“*I think that these multiprofessional groups*…*the HIV/AIDS services that have a good multiprofessional group*, *be it psychology*, *nursing*, *together with the doctor*, *social worker*, *ends up making a big difference*. *Often the doctor doesn’t understand*, *doesn’t see*, *I don’t know why*, *but sometimes we don’t see the whole picture*, *right*? *So these meetings help a lot*. *So I see that a patient who is going to be a candidate for chemoprophylaxis*, *he has to be seen by all the teams*, *right*? *So*, *in one of the outpatient clinics that I have here*, *there is a post-consultation*, *so nursing goes in with a social worker to assess the context a little*, *whether it [TPT] will work or not*.*”—Key Informant*, *Male*, *São Paulo*

Other suggestions, one especially prevalent among patients, included support groups for patients undergoing LTBI treatment using technology to maintain contact with patients about treatment, and involving family and/or friends in a patient’s treatment plan to provide additional adherence support. This included text-based reminder messages using locally popular communication platforms such as WhatsApp with specific suggestions to have these messages come from HCPs to their patients for further impact.

## Discussion

In this in-depth qualitative study conducted among HCPs, KIs and PLHIV in three cities in Brazil, knowledge, motivation, and support for LTBI screening and treatment were often constrained by logistical, operational and structural factors across clinical settings. Despite these challenges, providers and patients were open and eager for information, training and new technologies that might help address these constraints. Implementation science research, such as the qualitative research conducted in this study, allows us to develop the evidence base that informs the effective adoption of these new technologies into routine health care [25]. Understanding the acceptability and feasibility of LTBI screening and treatment tools in real world settings from patient and provider perspectives will inform optimal roll-out of these interventions.

Novel technologies for LTBI screening and treatment such as QFT+ and 3HP were seen as logistically advantageous over TST and current 6- or 9-month TPT regimen for PLHIV by a wide majority of participants. Findings indicate that these interventions have a strong potential to streamline many of the current challenges in LTBI screening and treatment, but they are not a “magic bullet”, as their implementation may require additional interventions to ensure equitable access and uptake in PLHIV.

Current WHO recommendations note that the absence of a positive LTBI test should not preclude TPT scale-up in PLHIV due to the feasibility of large-scale testing in burdened health systems and structural constraints [26]. However, it is likely that many moderate burden countries like Brazil will continue to recommend LTBI testing for PLHIV. In turn, to facilitate the use of novel technologies such as QFT+ and 3HP to reduce TB mortality among PLHIV, multi-level interventions must be developed, implemented and evaluated in parallel to the consideration of this new WHO recommendation. Our findings point to the need for health education about LTBI for health professionals, and improved communication between providers and patients about the risks of LTBI. In addition to individual level supports, logistical and structural intervention elements will also be needed. The overburdened health system and under-resourced and under-staffed clinics in Brazil (which are reflective of many resource-constrained settings with high TB burdens) underpin the low motivation to screen and treat LTBI. Hence, addressing these gaps will be critical to ensuring the successful implementation of QFT+ and 3HP. In addition, the support of and investment in HIV civil society organizations to offer and implement complementary interventions to improve uptake of and adherence to these biomedical interventions will be critical to their success.

Our findings suggest that these multi-level interventions should include attention to: (1) health education and training at the individual level; (2) supply chains, increased staffing, and monitoring and follow up of patient LTBI screening and treatment protocols at the clinic level; (3) access to health care providers via online communication platforms (e.g. WhatsApp) (4) laboratory protocols and assurances that results are available and utilized in a time sensitive manner at the operational levels; and (5) attention to the structural constraints and realities facing patients, such as food security, transportation, time off from work and child care needs. Such multi-level interventions that increase understanding of the importance of LTBI and TPT among HCPs, improve patient-provider communication and patient literacy, and streamline clinic-level operations related to QFT+ and 3HP use will enhance their optimization and in turn increase the possibility for significant reductions in TB-related mortality among PLHIV globally.

## References

[1] Joint United Nations Programme on HIV/AIDS. GLOBAL AIDS UPDATE 2016 [Internet] Geneva, Switzerland: UNAIDS; 2016. Available from: http://pesquisa.bvsalud.org/portal/resource/pt/mdl-15080170.

[2] World Health Organization. Global tuberculosis report 2021 [Internet] Geneva: WHO; 2021 [cited 2022 Mar 1]. Available from: https://apps.who.int/iris/handle/10665/346387.

[3] Ministério da Saúde, Secretaria de Vigilância em Saúde, Departamento de Vigilância das Doenças Transmissíveis. Brasil Livre da Tuberculose: Plano Nacional pelo Fim da Tuberculose como Problema de Saúde Pública. Brasília Ministério da Saúde, 2017.

[4] Coelho De Brito A, Alves Da Silva D, Gomes D OD, Arakaki D DF, al. e. Boletim Epidemiológico de Tuberculose: Ministério da Saúde; 2021 [cited 2022 Mar 17]. Available from: https://www.gov.br/saude/pt-br/media/pdf/2021/marco/24/boletim-tuberculose-2021_24.03.

[5] Ministério da Saúde, Secretaria de Vigilância em Saúde, Departamento de Vigilância das Doenças Transmissíveis. Protocolo de vigilância da infecção latente pelo Mycobacterium tuberculosis no Brasil. Brasília: Ministério da Saúde, 2018.

[6] Yamasue M, Komiya K, Usagawa Y, Umeki K, Nureki SI, Ando M, et al. Factors associated with false negative interferon-γ release assay results in patients with tuberculosis: A systematic review with meta-analysis. Sci Rep. 2020;10(1):1607. Epub 2020/02/02. doi: 10.1038/s41598-020-58459-9 ; PubMed Central PMCID: PMC6994686. 32005930 PMC6994686

[7] World Health Organization. Global Tuberculosis Report, Geneva, Switzerland: WHO; 2017 [cited 2021 Aug 23]. Available from: http://www.who.int/tb/publications/global_report/en/ [Google Scholar].

[8] Pathmanathan I, Ahmedov S, Pevzner E, Anyalechi G, Modi S, Kirking H, et al. TB preventive therapy for people living with HIV: key considerations for scale-up in resource-limited settings. Int J Tuberc Lung Dis. 2018;22(6):596–605. Epub 2018/06/05. doi: 10.5588/ijtld.17.0758 ; PubMed Central PMCID: PMC5989571. 29862942 PMC5989571

[9] Lalvani A, Pareek M. Interferon gamma release assays: principles and practice. Enferm Infecc Microbiol Clin. 2010;28(4):245–52. Epub 2009/09/29. doi: 10.1016/j.eimc.2009.05.012 . 19783328

[10] Secretaria Municipal de Saude. Diagnóstico e Tratamento da Infecção Latente por Tuberculose—ILTB [Internet] 2020 [cited 2022 Mar 17]. Available from: https://www.prefeitura.sp.gov.br/cidade/secretarias/upload/saude/nota_informativa_05_2020_diag_trat_tuberculose.pdf.

[11] Paim J, Travassos C, Almeida C, Bahia L, Macinko J. The Brazilian health system: history, advances, and challenges. Lancet. 2011;377(9779):1778–97. Epub 2011/05/13. doi: 10.1016/S0140-6736(11)60054-8 . 21561655

[12] Stuurman AL, Vonk Noordegraaf-Schouten M, van Kessel F, Oordt-Speets AM, Sandgren A, van der Werf MJ. Interventions for improving adherence to treatment for latent tuberculosis infection: a systematic review. BMC Infect Dis. 2016;16:257. Epub 2016/06/09. doi: 10.1186/s12879-016-1549-4 ; PubMed Central PMCID: PMC4897858. 27268103 PMC4897858

[13] Martinson NA, Barnes GL, Moulton LH, Msandiwa R, Hausler H, Ram M, et al. New regimens to prevent tuberculosis in adults with HIV infection. N Engl J Med. 2011;365(1):11–20. Epub 2011/07/08. doi: 10.1056/NEJMoa1005136 ; PubMed Central PMCID: PMC3407678. 21732833 PMC3407678

[14] Walker RE, Bass S, Srinivas P, Miranda C, Johnson L, Pallotta AM. Evaluation of 3 Months of Once-Weekly Rifapentine and Isoniazid for Latent Tuberculosis Infection. Ann Pharmacother. 2020;54(5):457–63. Epub 2019/11/16. doi: 10.1177/1060028019888855 . 31729245

[15] Pease C, Hutton B, Yazdi F, Wolfe D, Hamel C, Quach P, et al. Efficacy and completion rates of rifapentine and isoniazid (3HP) compared to other treatment regimens for latent tuberculosis infection: a systematic review with network meta-analyses. BMC Infect Dis. 2017;17(1):265. Epub 2017/04/13. doi: 10.1186/s12879-017-2377-x ; PubMed Central PMCID: PMC5387294. 28399802 PMC5387294

[16] Sterling TR, Villarino ME, Borisov AS, Shang N, Gordin F, Bliven-Sizemore E, et al. Three months of rifapentine and isoniazid for latent tuberculosis infection. N Engl J Med. 2011;365(23):2155–66. Epub 2011/12/14. doi: 10.1056/NEJMoa1104875 . 22150035

[17] Huang YW, Yang SF, Yeh YP, Tsao TC, Tsao SM. Impacts of 12-dose regimen for latent tuberculosis infection: Treatment completion rate and cost-effectiveness in Taiwan. Medicine (Baltimore). 2016;95(34):e4126. Epub 2016/08/26. doi: 10.1097/md.0000000000004126 ; PubMed Central PMCID: PMC5400306. 27559940 PMC5400306

[18] Maciel EL, Prado TND, Andrade KB, Golub JE. Is it time for Brazil to prioritize TB preventive therapy for all people living with HIV? Braz J Infect Dis. 2018;22(1):74–5. Epub 2017/11/10. doi: 10.1016/j.bjid.2017.10.001 ; PubMed Central PMCID: PMC9425644. 29121486 PMC9425644

[19] Semitala FC, Kadota JL, Musinguzi A, Nabunje J, Welishe F, Nakitende A, et al. Completion of isoniazid-rifapentine (3HP) for tuberculosis prevention among people living with HIV: Interim analysis of a hybrid type 3 effectiveness-implementation randomized trial. PLoS Med. 2021;18(12):e1003875. Epub 2021/12/17. doi: 10.1371/journal.pmed.1003875 ; PubMed Central PMCID: PMC8726462 following competing interests: AS receives research funding from Global Health Labs, California Health Care Foundation and is owner of The Empathy Studio, LLC. DP is a human-centered design consultant for The Empathy Studio, LLC. The other authors have declared that no competing interests exist. 34914696 PMC8726462

[20] Cui X, Gao L, Cao B. Management of latent tuberculosis infection in China: Exploring solutions suitable for high-burden countries. Int J Infect Dis. 2020;92s:S37-s40. Epub 2020/03/03. doi: 10.1016/j.ijid.2020.02.034 . 32114201

[21] Yuen CM, Majidulla A, Jaswal M, Safdar N, Malik AA, Khan AJ, et al. Cost of Delivering 12-Dose Isoniazid and Rifapentine Versus 6 Months of Isoniazid for Tuberculosis Infection in a High-Burden Setting. Clin Infect Dis. 2021;73(5):e1135–e41. Epub 2020/12/09. doi: 10.1093/cid/ciaa1835 ; PubMed Central PMCID: PMC8423476. 33289039 PMC8423476

[22] Keddem S, Barg FK, Frasso R. Practical Guidance for Studies Using Freelisting Interviews. Prev Chronic Dis. 2021;18:E04. Epub 2021/01/15. doi: 10.5888/pcd17.200355 ; PubMed Central PMCID: PMC7845553. 33444525 PMC7845553

[23] Boyatzis R. Transforming qualitative information: Thematic analysis and code development. Thousand Oaks, CA: SAGE Publications; 1998.

[24] Mulhall A. In the field: notes on observation in qualitative research. J Adv Nurs. 2003;41(3):306–13. Epub 2003/02/13. doi: 10.1046/j.1365-2648.2003.02514.x . 12581118

[25] Allotey P, Reidpath DD, Ghalib H, Pagnoni F, Skelly WC. Efficacious, effective, and embedded interventions: Implementation research in infectious disease control. BMC Public Health. 2008;8(1):343. doi: 10.1186/1471-2458-8-343 18826655 PMC2567977

[26] World Health Organization. WHO consolidated guidelines on tuberculosis: module 1: prevention: tuberculosis preventive treatment [Internet] Geneva: World Health Organization; 2020 [cited 2022 Mar 15]. Available from: https://apps.who.int/iris/handle/10665/331170.

